# Rapid detection of isocitrate dehydrogenase 1 mutation status in glioma based on Crispr-Cas12a

**DOI:** 10.1038/s41598-023-32957-y

**Published:** 2023-04-07

**Authors:** Zhebin Feng, Dongsheng Kong, Wei Jin, Kunyu He, Junyan Zhao, Bin Liu, Hanyun Xu, Xin’guang Yu, Shiyu Feng

**Affiliations:** Senior Department of Neurosurgery, The First Medical Center of PLA General Hospital, 28 Fuxing Road, Haidian District, Beijing, 100089 China

**Keywords:** Biological techniques, Neurology

## Abstract

The aim is to use Crispr-Cas12a for the rapid detection of the single nucleotide polymorphism (SNP) of isocitrate dehydrogenase 1 (IDH1)-R132H locus and explore the effectiveness and consistency of this method with direct sequencing method for detecting IDH1-R132H of glioma tissue samples. 58 previous frozen tissue and 46 recent fresh tissue samples of adult diffuse glioma were selected to detect IDH1-R132H using Crispr-Cas12a. The results of immunohistochemistry (IHC) and direct sequencing methods were analyzed. We calculated the efficiency index of Crispr-Cas12a and IHC, and analyzed the consistency among Crispr-Cas12a, IHC and direct sequencing method using paired Chi-sequare test and Kappa identity test. We accomplished the rapid detection of IDH1-R132H in 60 min using Crispr-Cas12a. Regarding direct sequencing method as the gold standard, the sensitivity, specificity and consistency rate of Crispr-Cas12a was 91.4%, 95.7% and 93.1% in the frozen sample group, while 96.1%, 89.7% and 92.0% in the fresh sample group, respectively. Kappa test showed good consistency between the two methods (k = 0.858). Crispr-Cas12a can quickly and accurately detect IDH1-R132H and has good stability. It is a promising method to detect IDH1 mutation status intraoperatively.

## Introduction

Glioma is the most common intracranial primary malignancy with an annual incidence of (5–6)/100,000 worldwide^1^. At present, the preferred treatment for glioma is maximal safe resection with functional preservation^2,3^. The surgery can significantly prolong the progression-free survival (PFS) and the overall survival (OS) time of patients^4,5^. However, due to the characteristics of extensive invasion and infiltrative growth of gliomas, tumors often have no distinct boundary with the surrounding normal brain tissues^6^, which poses a great challenge for the determination of resection range. For now, there is no common theory about the excision extension for different subtypes of glioma. For histopathological classification, the formulation of glioma surgical strategy also depends on the molecular characteristics such as isocitrate dehydrogenase (IDH), ATRX, TERT, chromosome 1p/19q^7^, while IDH is one of the most important molecular biomarkers of glioma^8^. Among them, IDH1-R132H mutation usually occurs in low-grade gliomas^9^. Preoperative or intraoperative knowledge of IDH molecular status is of great significance for personalized surgery^10^. Therefore, a simple, rapid and biosafe Point Of Care Testing (POCT) technology for IDH molecular status is an unmet need and is of great public health importance.

Clustered regularly interspaced short palindromic repeats (Crispr) is a stretch of repetitive sequence in the prokaryotic genome, and it together with Crispr-associated (Cas) protein constitutes the Crispr-Cas system, which uses RNA-mediated nucleases to degrade the target foreign nucleic acids^11,12^. As a new generation of detection technology, Crispr detection has been widely used in the field of medical detection and has achieved important results^13^. Among them, cas9 protein has received more attention in gene editing, while cas12a and cas13a are more efficient in disease diagnosis^14^. Cas12a is an RNA-directed endonuclease, which is guided by a self-derived RNA (Crispr RNA, crRNA) that is complementary to the target double-stranded DNA (dsDNA). The enzyme presents robust and undifferentiated activity of cutting single-stranded DNA (ssDNA) after binding to the target dsDNA. When cleaving the dsDNA (which called "cis cleavage"), it triggers the collateral trans cleaving of short single-stranded DNA (ssDNA) and allows for the separation of the fluorophore from the quencher of the reporter group, resulting in the fluorescence^15,16^. Crispr-Cas12a protein has been used in clinical diagnosis in many fields and directions. Liang et al. described and analytically validated a Crispr-Cas12a-based gene-specific testing method that could be used for rapid detection of SARS-CoV-2 VOCs^17^. Oraphan Mayuramart et al. applied the Crispr-Cas12-based technology to the detection of SARS-CoV-2 and influenza viruses to achieve effective screening of COVID-19 and influenza virus-infected patients^18^. In addition, Qian et al. developed a detection technique combining reverse transcriptase recombinase polymerase amplification (RT-RPA) with Crispr-Cas12a (RT-RPA-Cas12a) for clinical diagnosis of human metapneumovirus (HmPV)^19^.

Here we verified the feasibility of a POCT of the IDH1-R132H (c.395G>A) in frozen and fresh glioma tissue samples based on Crispr-Cas12a. It is expected to provide a new mean to achieve "maximum safe excision" along the "molecular boundary".

## Materials and methods

### Main reagents and instruments

HiScribe T7 Quick High Yield RNA Synthesis kit (NEB, Ipswich, United States), TRIeasy™ Total RNA Extraction Reagent (Yisheng Biotechnology (Shanghai) Co., Ltd, China), Fncas12a (Jiangsu Bojia Biomedical Technology Co., LTD, China), V1 palmtop fast fluorescence detector (Jiangsu Bojia Biomedical Technology Co., LTD, China), water bath and high speed refrigerated centrifuge were used.

### Sample source and inclusion and exclusion criteria

62 frozen samples of adult diffuse gliomas, which were removed by craniocerebral operation and preserved in tumor tissue bank, were selected from 2019 to 2020 in the Department of Neurosurgery, Chinese PLA General Hospital, including 40 IDH1-R132H cases and 22 IDH1-wt cases (Group 1). In addition, 48 fresh samples of newly diagnosed and consecutive brain tumor cases were suspected as adult diffuse glioma according to the clinical manifestation and the imaging examination before the operation being collected from November 2021 to March 2022 (Group 2). Fresh samples were sampled intraoperatively at the center of the tumors. More malignant areas had the higher spectral Cho/NAA peak, enhanced lesions in MRI (magnetic resonance imaging) and higher perfusion with the assistance of neuron avigation system. Meanwhile, we tried to avoid the areas like stroke, necrosis, secretory capsule, edema and tumor margin as much as possible.

For previous frozen samples, we numbered them and used RAND function to generate random numbers for each sample in Excel. The Crispr detection order was determined according to the size of random number and the inspectors weren't aware of the status of IDH. As for recent fresh samples, the Crispr detection order was determined according to the chronological order, which was completed before the results of IHC and direct sequencing method being obtained. The procedures above all met the blinded requirements.

All data were typed into the statistical software twice. Crispr-Cas12a was repeated three times per sample parallelly. The IHC and direct sequencing method (Sanger sequencing or second-generation sequencing method) referred to the results of the patients’ postoperative test.

Inclusive criteria: (1) Over 18 years old and postoperative pathology confirmed that the diagnosis was adult diffuse glioma, WHO grade 2 to 4 (refer to the 2021 WHO classification of tumors of central nervous system^7^); (2) Tissue samples were placed in cryogenic vials within 24 h after surgery and stored in liquid nitrogen without freeze–thaw before Crispr detection; (3) The patient or his or her families signed the informed consent. Exclusive criteria: (1) Suffering from other malignant tumors; (2) Accept radio chemotherapy, tumor treating fields, biological therapy for glioma before surgery; (3) Accompanied by serious bacterial, fungal, viral infectious diseases and other infectious diseases. The demographic data of enrolled patients were shown in Table 1.

**Table 1:** Demographic data of enrolled patients.

	IDH1-wt	IDH1-R132H	Value (P)	Difference (95% CI)	Total
Age at diagnosis, mean (SD)	55.58 ± 17.77	44.2 ± 12. 18	Z = − 2.90 (**0.004**)	16 (9–23)	48.98 ± 14.37
Sex, n (%)			Χ^2^ = 0.036 (0.851)	0.025 (− 0.219 to 0.266)	
Male	11 (50)	19 (47.5)			30 (48.4)
Female	11 (50)	21 (52.5)			32 (51.6)
Location, n (%) *			Χ_c_^2^ = 15.07 (**0.005**)		
Frontal lobe	7 (31.8)	32 (80.0)	Χ^2^ = 14.12 (< **0.001**)	0.482 (0.226 to 0.663)	39 (62.9)
Temporal lobe	10 (45.5)	5 (12.5)	Χ^2^ = 8.405(**0.004**)	0.330 (0.100 to 0.541)	15 (24.2)
Parietal lobe	2 (9.1)	2 (5.0)	– (0.61)	0.041_c_ (− 0.111 to 0.260)	4 (6.5)
Occipital lobe	1 (4.5)	0 (0.0)	– (0.36)	0.046_c_ (− 0.072 to 0.249)	1 (1.6)
Insular lobe	2 (9.1)	1 (9.1)	– (0.28)	0.066_c_ (− 0.078 to 0.284)	3 (4.8)
			Χ_c_^2^ = 0.026 (0.987)		
Left cerebral hemisphere	9 (40.9)	17 (42.5)	Χ^2^ = 0.015 (0.903)	0.016 (− 0.231 to 0.250)	26 (41.9)
Right cerebral hemisphere	12 (54.5)	21 (52.5)	Χ^2^ = 0.024 (0.877)	0.021 (− 0.226 to 0.259)	33 (53.2)
Both right and left cerebral hemisphere	1 (4.5)	2 (5.0)	– (1.00)	0.005_c_ (− 0.20 to 0.14)	3 (4.8)
Histopathology, n (%)			Χ_c_^2^ = 14.12 (< **0.001**)		
Oligodendroglioma	2 (9.1)	26 (65.0)	Χ^2^ = 17.914 (< **0.001**)	0.559 (0.316 to 0.704)	28 (45.2)
Astrocytoma	2 (9.1)	7 (17.5)	Χ_c_^2^ = 0.273 (0.601)	0.084_c_ (− 0.152 to 0.260)	9 (14.5)
Glioblastoma	18 (81.8)	6 (15.0)	Χ^2^ = 26.710 (< **0.001**)	0.668 (0.421 to 0.803)	24 (38.7)
Not classification	0 (0.0)	1 (2.5)	– (1.00)	0.025_c_ (− 0.162 to 0.147)	1 (1.6)
WHO classification, n (%)			– (< **0.001**)		
Grade 2	0 (0.0)	11 (27.5)	– (**0.005**)	0.275_c_ (0.053 to 0.441)	11 (17.7)
Grade 3	4 (18.2)	23 (57.5)	Χ^2^ = 8.925 (**0.003**)	0.393 (0.139 to 0.570)	27 (43.5)
Grade 4	18 (81.8)	6 (15.0)	Χ^2^ = 26.71 (< **0.001**)	0.668 (0.421 to 0.803)	24 (38.7)

	IDH1-wt	IDH1-R132H	Value (P)	Difference (95% CI)	Total
Age at diagnosis, mean (SD)	54.96 ± 11.26	44 ± 10.48	Z = − 3.26 (**0.001**)	14 (7–19)	50.43 ± 12.13
Sex, n (%)			Χ^2^ = 0.199 (0.766)	0.066 (− 0.207 to 0.331)	
Male	16 (59.3)	10 (52.6)			26 (56.5)
Female	11 (40.7)	9 (47.4)			20 (43.5)
Location, n (%) *			– (**0.002**)		
Frontal lobe	8 (29.6)	11 (57.9)	Χ^2^ = 3.675 (0.073)	0.283 (− 0.004 to 0.517)	19 (41.3)
Temporal lobe	11 (40.7)	1 (5.3)	Χ_c_^2^ = 5.556 (**0.018**)	0.355_c_ (0.066 to 0.563)	12 (26.1)
Parietal lobe	3 (11.1)	3 (15.8)	Χ_c_^2^ = 0.000 (0,985)	0.047_c_ (− 0.178 to 0.307)	6 (13.0)
Occipital lobe	3 (11.1)	0 (0.0)	– (0.257)	0.111_c_ (− 0.114 to 0.303)	3 (6.5)
Insular lobe	0 (0.0)	4 (21.1)	– (**0.024**)	0.211_c_ (0.033 to 0.433)	4 (8.7)
Others	2 (7.4)	0 (0.0)	– (0.504)	0.074_c_ (− 0.144 to 0.258)	2 (4.3)
			– (0.286)		
Left cerebral hemisphere	11 (40.7)	12 (63.2)	Χ^2^ = 2.242 (0.231)	0.224 (− 0.064 to 0.464)	23 (50.0)
Right cerebral hemisphere	15 (55.6)	7 (36.8)	Χ^2^ = 1.565 (0.245)	0.187 (− 0.100 to 0.432)	22 (47.8)
Both right and left cerebral hemisphere	1 (3.7)	0 (0.0)	– (1.000)	0.037_c_ (− 0.175 to 0.209)	1 (2.2)
Histopathology, n (%)			– (< **0.001**)		
Oligodendroglioma	2 (7.4)	9 (47.4)	Χ_c_^2^ = 7.715 (**0.005**)	0.400_c_ (0.112 to 0.639)	11 (23.9)
Astrocytoma	2 (7.4)	9 (47.4)	Χ_c_^2^ = 7.715 (**0.005**)	0.400_c_ (0.112 to 0.639)	11 (23.9)
Glioblastoma	20 (74.1)	0 (0.0)	– (< **0.001**)	0.741_c_ (0.489 to 0.868)	20 (43.5)
Not classification	3 (11.1)	1 (5.3)	Χ_c_^2^ = 0.026 (0.872)	0.059_c_ (− 0.184 to 0.257)	4 (8.7)
WHO classification, n (%)			Χ_c_^2^ = 15.557 (**0.001**)		
Grade 2	4 (14.8)	8 (42.1)	Χ_c_^2^ = 3.009 (0.083)	0.273_c_ (− 0.016 to 0.532)	12 (26.1)
Grade 3	2 (7.4)	7 (36.8)	Χ_c_^2^ = 4.412 (**0.036)**	0.294_c_ (0.026 to 0.547)	9 (19.6)
Grade 4	21 (77.8)	4 (21.0)	Χ^2^ = 14.463 (< **0.001)**	0.567 (0.277 to 0.738)	25 (54.3)

Significant values are in bold.

### Reagent preparation and system verification

#### Primer design and preparation of crRNA

IDH1-R132H (c.395G>A) was selected as the detective locus. IDH1-wt and IDH1-R132H were designed with reference to the known mutation detection loci for the specific gene. Amplimers and crRNAs were then designed for the known mutation regions. This crRNA guided the mutant target to bind to cas12a protein to form a trijunction complex to activate the disordered cleavage activity of the protein. When cutting the target, a fluorescence probe could be cut to transmit fluorescence reading signals. However, if there is no mutant sequence in the target, the crRNA and protein cannot be bound, so the cleavage activity of the protein will not be activated, and the strong fluorescence signal will not be generated. And oligonucleotides (crDNA) were synthesized. CrDNA and T7-crRNA-F were mixed, and the double-stranded transcription templates were formed by annealing the double-stranded target sequences (100 °C, 10 min, then natural cooling). The transcription template was incubated for 16 h at 37 °C under enzymatic-free conditions using the HiScribe T7 Quick High Yield RNA Synthesis Kit (NEB, Ipswich, United States). After the completion of the reaction, 2 μL of DNase 1 (TianGen, Beijing, China) was added to eliminate unreacted template and the crRNA was purified. IDH1-wt and IDH1-R132H sequences, amplimers and crDNAs were synthesized by Suzhou Jinweizhi Biotechnology Co., Ltd. CrDNA, primers and information of IDH1-wt and IDH1-R132H sequences were shown in Table 2.

**Table 2:** crDNA, upsteam and downsteam primer, IDH1-wt and IDH1-R132H sequences.

Subjects	Sequences
IDH1-crDNA	5′-GGCATCATGCTTATGGGGATATCTACAAGAGTAGAAATTACCCTATAGTGAGTCGTATTAATTTC-3′
IDH1-F primer	5′-GCAGAAGCTATAAAGAAGCATAATGTTG-3′
IDH1-R primer	5′-AAATCACATTATTGCCAACATGACTTAC-3′
T7 promoter	5′-GAAATTAATACGACTCACTATAGGG-3′
IDH1-wt	5′-TTACTTGATCCCCATAAGCATGA**C**GACCTATGATGATAGGTTTTACCCATCCACTCACAAGCCG-3′
IDH1-R132H	5′-TTACTTGATCCCCATAAGCATGA**T**GACCTATGATGATAGGTTTTACCCATCCACTCACAAGCCG-3′
ssDNA	FAM-CCCCCC-BHQ1

#### Amplification system and conditions

Follow the program: 95 °C, 180 s; 95 °C, 10 s; 58 °C, 20 s; 30 circles; 25 °C, 2 s.

#### Validation of Crispr-Cas12a fluorescence detection system

The detective protein is Fncas12a, which used 5′-KYTV-3′ as protospacer adjacent motif (PAM). The syntheized target template was added into the detection reagent mixture (including 0.75 μM crRNA, 1.5 μM Fncas12a, 50 pM of fluorescent probe and 3 μL of NEBuffer 2.1) (Table 3), then incubated at 37 °C for around 30 min. Then read the fluorescence quantification value and analyze the curve. All reactions were carried out at 37 °C. The fluorescent probe was generic, not specific to IDH1-wt and IDH1-R132H, but labeled to FAM fluorophores. The key in the system is the crRNA, not the probe. FAM is a purified single isomer of carboxyfluorescein. It is one of the most popular green fluorescent reagents used to label peptides, proteins and nucleotides. It has an excitation wavelength of 495 nm and an emission wavelength of 518 nm.

**Table 3:** The fluorescence detection system of Crispr-Cas12a.

Subjects	Volume
Fluorescence probe	50 pm
Target DNA template	1–5 μL
crRNA	0.75 µM
FnCas12a	1.5 µM
NEBuffer2.1	3 μL
DEPC-water	100 μL

### Detection of clinical samples

#### Processing of samples

Cut or suck a little tumor fresh or frozen tissue (about 0.1 mL), melt it at room temperature and use rapid DNA extraction detection kit (Tianyuan Biology). Add A1 Buffer 50 μL, incubate at room temperature for 10 min after sufficiently grinding and shaking the tissues, then add A2 Buffer 50 μL and centrifuge the mixture.

#### Reagent preparation

PCR was performed using conventional primers. 5 μL sample supernate was used as template. Add the amplification system into the upper tube (storage tube) of reaction consumables and throw it into the lower tube (reaction tube) through the designed connected device on the consumables. After that, switch the device to the lock position and disconnect the upper and lower tube. A total of 50 μL mixture of the detection system (which containing purified Cas12a, crRNA, fluorescent probe, DNA enzyme inhibitor and enzyme-free water) was added to the upper reservoir tube of consumables.

### Amplification of target gene and Crispr-Cas12a fluorescence detection

**Figure 1:**
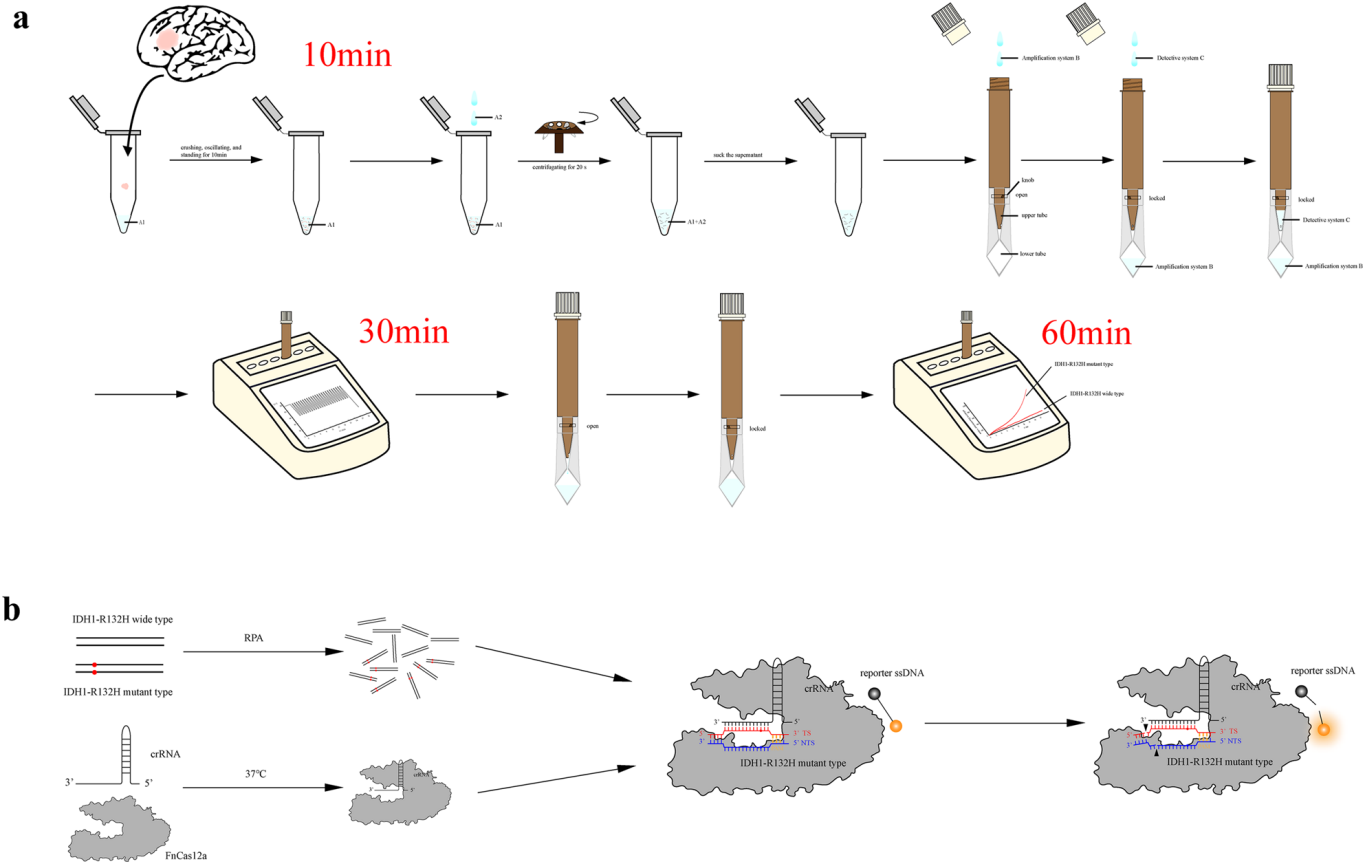
Establishment of Crispr-Cas12a fluorescence detection system. (**a**) Schematic diagram of the Crispr for IDH1-R132H detection. Nucleic acid extraction, detection, result determination and reporting were all integrated in a single instrument, the whole process could obtain test results in 60 min. (**b**) The Crispr detection system for IDH1-R132H. The change of the fluorescence value was recorded at the end of the reaction, and the IDH1-R132H was determined according to the threshold of the fluorescence value (link: https://www.adobe.com/products/illustrator.html/, version 24.0.1).

V1 palmtop fast fluorescent detector (Jiangsu Bojia Biomedical Technology Co., LTD, China) was used for detection. The expansion followed the procedure: 95 °C, 180 s; 95 °C, 10 s, 58 °C, 20 s, 23 cycles; 25 °C, 2 s. After the amplification reaction, the detection mixture was thrown into the lower tube through the connected device and the tube was inserted to the slot of the fluorescent detector again. The reaction program: 37 °C, 1 min, 30 cycles, and the fluorescence was collected once a minute. The change of the fluorescence value was recorded at the end of the reaction, and the status of IDH1 was determined according to the threshold of the fluorescence value (Fig. 1).

### Other detective methods

All patients were tested for IHC (EnVision method) and direct sequencing method. IHC was completed by the Department of Pathology, The First Medical Center of the PLA General Hospital. For IHC method, staining of more than 10% tumor cells was seen as IDH1-R132H, the spotted, suspicious and weak staining of the tumor cells was seen as IDH1-wt. Direct sequencing method which used one-step method (multiplex PCR amplification combined with next-generating sequencing) was completed by Genetron Health Inc., Beijing, China. 62 previous frozen samples were tested additionally for droplet digital PCR (ddPCR) and HE staining to verify the quality of frozen samples. We regarded the conjoint result of direct sequencing method and ddPCR as the gold standard and evaluated the accuracy of Crispr detection.

### Results of analysis and statistical evaluation of Crispr-Cas12a

Statistical analysis was performed using SPSS 22.0 software, the 95% credible interval of the rate was aided by Vassarstats (http://vassarstats.net/index.html). Regard the conjoint result of direct sequencing method and ddPCR as the gold standard and evaluate the accuracy of Crispr detection. The receiver operating characteristic (ROC) curve was drawn and the area under curve (AUC) was calculated. The optimal cut-off value for the relative fluorescence value of the IDH1-R132H and IDH1-wt was obtained. The sensitivity, specificity and consistency rate was calculated using fourfold table of diagnostic experiments. The differences and consistency in the IDH1-R132H detection were evaluated by paired chi-square test (McNemar test) and Kappa identity test (Kappa > 0.75 indicated a good agreement between the two methods, 0.4 ≤ Kappa < 0.75 was normal and Kappa < 0.4 was bad). *P* < 0.05 was considered to be statistically significant.

### Ethics statement

The experiments were approved by the Ethics Committee of the Chinese People’s Liberation Army General Hospital (Ethics Committee Approval No.: 2018 LUNSHEN No. 268, 2018 LUNSHEN No. 268-02). All the authors confirmed that all experiments were performed in accordance with relevant guidelines and regulations.

## Results

### Crispr-Cas12a detection of IDH1 mutations in glioma

Schematic of the operation of the CRISPR-Cas12a method. About 0.1 mL of frozen or fresh adult diffuse glioma tissue samples were placed in a centrifuge tube and 50μL of A1 Buffer was added. The tissue was thoroughly ground and crushed to a suspension using a glass rod or pipetting gun tip. If necessary, an oscillator can be used to speed up tissue mass dissolution. After standing for 10 min, A2 Buffer was added, and the supernatant was aspirated after shaking and centrifugation for further use. Assemble the direct expansion tube. The direct expansion tube is divided into two layers. The upper layer is a reservoir pipe with the function of avoiding light, which can preserve the reagent in the dark. The lower layer is a transparent reaction tube, which facilitates the machine to receive the fluorescence produced by the reaction. The two tubes are connected by a knob switch in the middle. When the knob is closed, the upper and lower tubes are not connected. When the knob is turned on, the liquid in the upper reservoir pipe can flow into or throw into the lower layer. The amplification system and detection system were configured as described above. During detection, turn on the knob, add amplification system first, and throw it into the lower layer of the straight expanding tube, then turn off the knob, add detection system and store it in the upper layer of the straight expanding tube, and cover the tube cover. The direct expansion tube was inserted into the corresponding hole position of the V1 handheld rapid fluorescence detector, and the PCR amplification was performed after setting the temperature, which took about 20 min. After amplification, turn on the knob, throw the upper tube detection system into the lower tube, so that the detection system and amplification products are mixed, and turn off the knob. The tube was inserted into the corresponding hole position of the V1 handheld rapid fluorescence detector, and the fluorescence reaction was carried out for 30 min after setting the temperature. The fluorescence value of the detector was read out in real time every minute, and the total time was about 60 min (Fig. 1a). Schematic diagram of the CRISPR-Cas12a fluorescence detection system. Under the corresponding amplification system, the amplified crRNA of tested DNA (including IDH1-R132H mutant and wild type sequences) was combined with FnCas12a enzyme at 37 °C. crRNA specifically recognized and bound to the IDH1-R132H mutant sequence. If there is an IDH1-R132H mutant sequence, FnCas12a enzyme activates the activity of cutting target dsDNA and ssDNA after the combination of the two, and the ssDNA connecting the fluorophore and the quenching agent is cut, so as to emit fluorescence, which is received by the fluorescence detector. If there is no IDH1-R132H mutant sequence, FnCas12a enzyme activity will not be activated, that is, there is no significant change in fluorescence value (Fig. 1b).

### WHO grade were associated with IDH1 status

**Figure 2:**
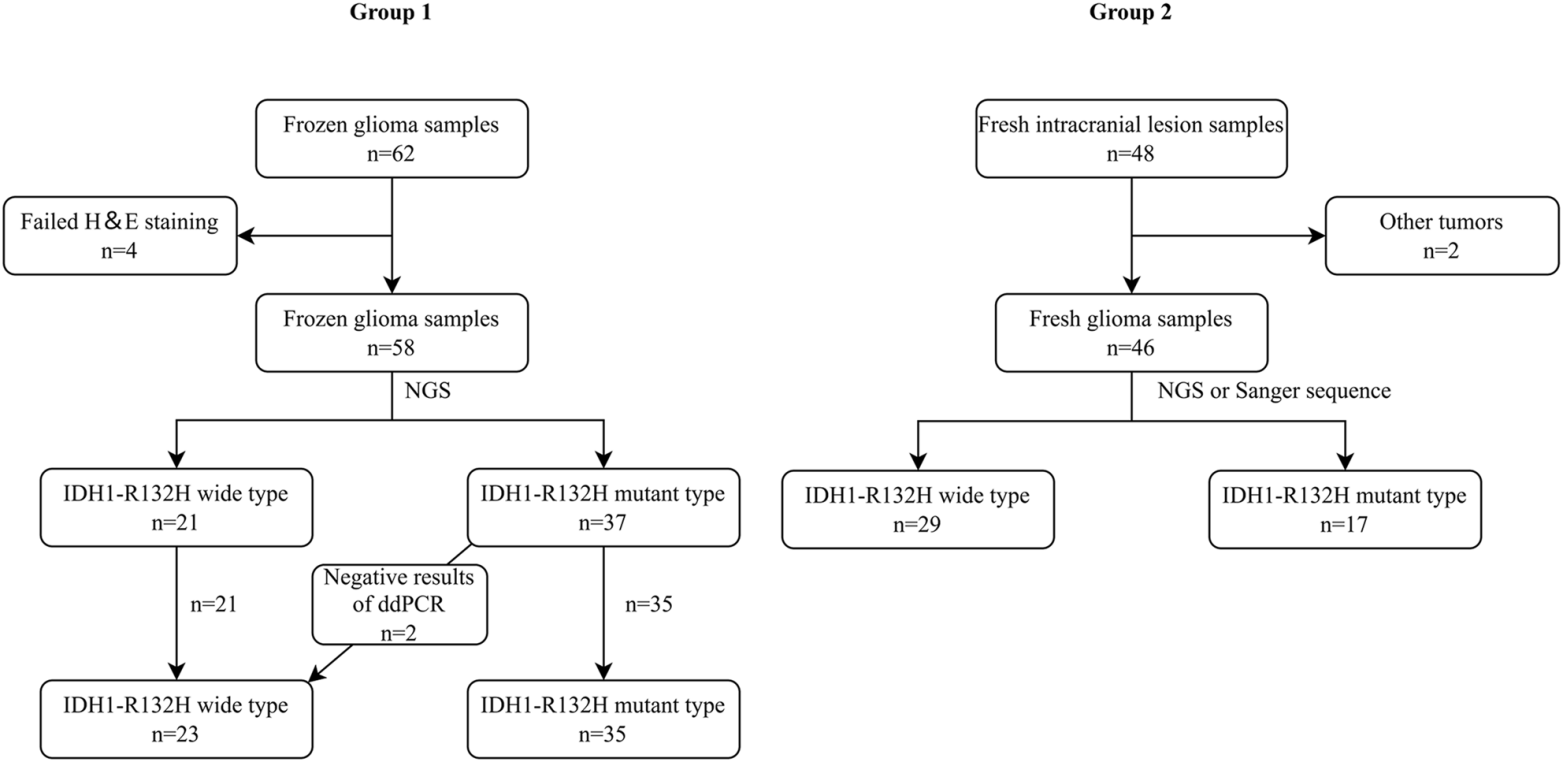
Distribution chart of IDH1-R132H in Group 1 and Group 2. A total of 110 clinical samples were selected; Group 1: 62 previous frozen glioma samples; Group 2: 48 recent fresh intracranial tumor samples. All patients were tested for IHC (EnVision method) and direct sequencing method. 62 previous frozen samples were tested additionally for droplet digital PCR (ddPCR) and HE staining to verify the quality of frozen samples. Four cases of HE staining failure and two cases of other tumors were excluded. We performed Crispr detection for 104 clinical samples. Then, the IDH1 mutation status of clinical samples was recorded. The concordance between Crispr-Cas12a, IHC, and direct sequencing methods was further analyzed (link: https://app.diagrams.net/, version 21.0.6).

Out of a total of 110 samples, there were 62 previous frozen glioma samples and 48 recent fresh intracranial tumor samples. The content of 4 samples of the tumor cells was less than 10% in Group 1, which was confirmed by HE staining. The above 4 samples were removed in the detection data analysis (Fig. 2). IDH1-R132H and IDH1-wt samples were 37 and 21 in Group 1, respectively. Two samples of Group 2 were not adult diffuse glioma, one was *pleomorphic xanthoastrocytoma*, WHO grade 2; and the other was renal cell carcinoma, which was confirmed by histology and pathology. The above 2 samples were removed in the detection data analysis. IDH1-R132H, IDH2-R172K, IDH1-I367V and IDH1-wt were 17, 1, 1, 27 in Group 2, respectively. Correlation analysis showed that the age, location of tumors, histopathology and WHO grade were associated with IDH1 status. Combined with molecular pathological conditions, 12 cases were improved diagnosis in Group 1, accounting for 19.4%. Detailed data of patients were shown in Table 1.

### High specificity and titer of Crispr-Cas12a for IDH1-R132H detection

**Figure 3:**
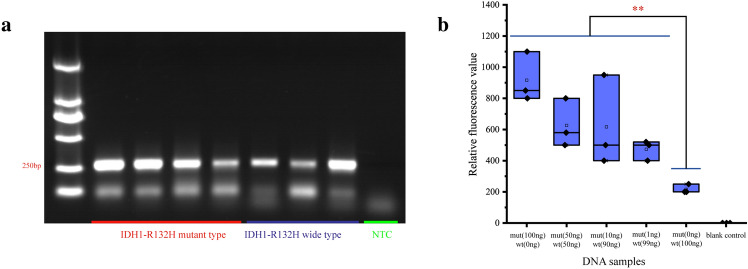
Validation of the Crispr-Cas12a fluorescence detection system. (**a**) Identification of the target gene amplification products by electrophoresis. The belts of IDH1-wt (the first four belts) and IDH1-R132H (the latter four belts) of electrophoresis with the length of 262 bp. (**b**) Verification of fluorescence system. Non-amplified IDH1-wt and IDH1-R132H were mixed in different proportions to simulate the IDH1-R132H at 100%, 50%, 50%, 10%, 1% and 0 frequencies in vivo, respectively. Using Crispr-Cas12a to perform the test for 3 times, the relative fluorescence value was positively correlated with the IDH mutation frequency range with qualitative detection power, and the lowest detectable mutation frequency was 1% theoretically (link: https://www.adobe.com/products/illustrator.html/, version 24.0.1).

The amplification of the target gene included the IDH1-R132H and IDH1-wt target gene with the target fragment length of 262 bp (Fig. 3a). Using different proportions of the mixture of IDH1-R132H and IDH1-wt to simulate IDH1-R132H at different frequencies, we found that the relative fluorescence value of Crispr detection was positively correlated with IDH1-R132H frequency generally, and the minimal detectable frequency of IDH1-R132H was 1% theoretically. The relative fluorescence cut-off value for IDH1-R132H and IDH1-wt was about 220 (Fig. 3b).

For ddPCR, 2 cases were inconsistent with the postoperative direct sequencing and IHC in Group 1 (Fig. 2). Both postoperative IHC and direct sequencing method showed IDH1 mutation, while ddPCR showed no mutations (0.08% and 0.11%, respectively). We regarded the samples of these 2 cases as IDH1-wt. Out of a total of 104 samples, only 1 case wasn’t coincident with direct sequencing method in Group 1 (Table 4). This astrocytoma (WHO grade 2) presented IDH1-wt in IHC while direct sequencing was shown as IDH1-R132H. The result of ddPCR was consistent with the mutation, and its frequency of mutation was 22%.

**Table 4:** Consistency analysis of two groups between IHC and direct sequencing method in IDH1-R132H detection.

IHC	Direct sequencing		Total
	+	−	
+	51	0	51
−	1	52	53
Total	52	52	104

### ROC curves and consistency analysis based on Crispr-Cas12a

**Figure 4:**
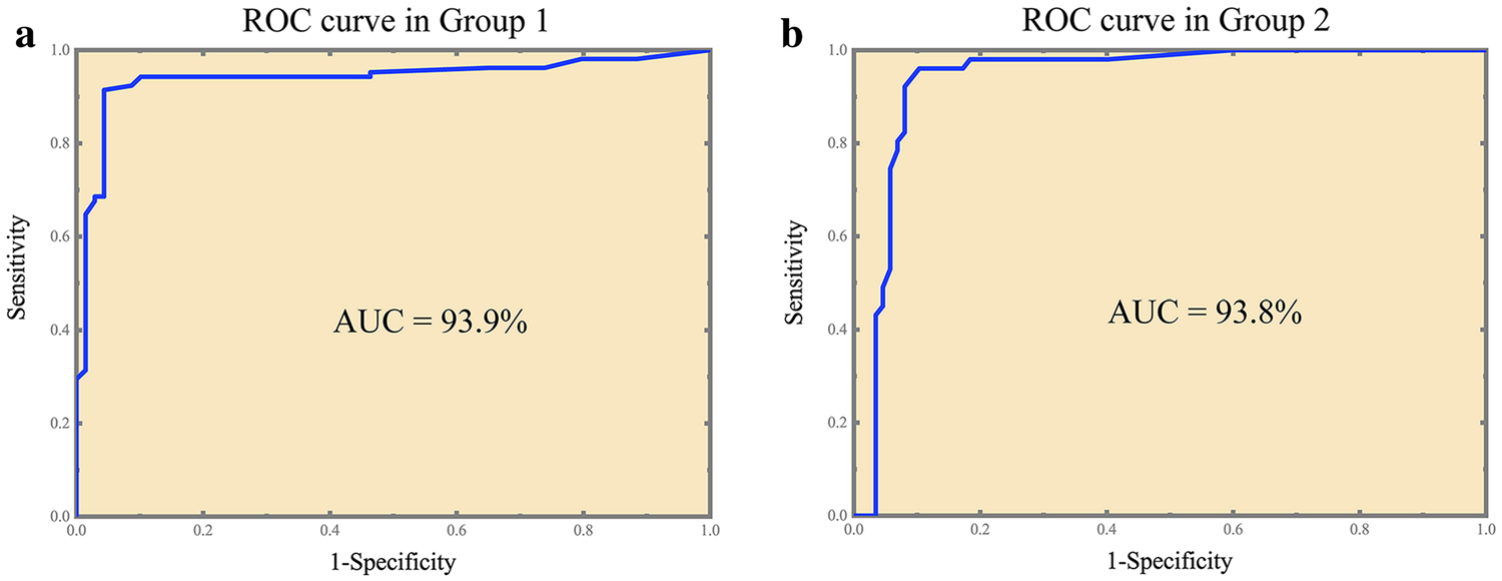
ROC curve of Crispr method. (**a**) ROC curve in Group 1. The value of AUC was 0.939 (95% CI 0.900–0.975); the threshold of relative fluorescence value was 425; the sensitivity, specificity and consistency rate was 91.4%, 95.7% and 93.1%, respectively. (**b**) ROC curve in Group 2. The value of AUC was 0.938 (95% CI 0.893–0.983); the threshold of relative fluorescence value was 525; the sensitivity, specificity and consistency rate was 96.1%, 89.7% and 92.0%, respectively (link: https://www.originlab.com/, version OriginPro 2018C).

First, we performed Crispr detection for 58 samples in Group 1 (23 IDH1-wt samples and 35 IDH1-R132H samples). The results of 3 replicates were consistent in 52 samples and inconsistent in 6 samples. 2 samples had one false negative result, 1 sample had two false negative results and 3 samples had one false positive result. We compared the results of Crispr detection of 174 cases with the gold standard and drew the ROC curve (Fig. 4a), and the value of AUC was 0.939 (95% CI 0.900–0.975). At this point, the threshold of relative fluorescence value was 425, and the sensitivity, specificity and consistency rate was 91.4% (95% CI 0.845–0.954), 95.7% (95% CI 0.880–0.985) and 93.1% (95% CI 0.883–0.960), respectively (Table 5). Youden index was 0.871 and McNemar test showed that there was no statistical difference between Crispr method and the direct sequencing method. The Kappa value was 0.858, which showed good consistency with the gold standard. There was also no statistical difference between the IHC method and the gold standard. The Kappa value was 0.965, which indicated that IHC had the good consistency with the direct sequencing method.

**Table 5:** Consistency analysis of cryopreserved sample groups between Crispr and direct sequencing method in IDH1-R132H detection.

Crispr	Gold standard		Total
	+	−	
+	96	3	99
−	9	66	75
Total	105	69	174

Next, we analyzed the results of Crispr detection of 46 samples in Group 2 (Fig. 4b). According to the threshold of Group 1 (relative fluorescence value was 425), the results showed a sensitivity of 98.0% and a specificity of 81.6% at this threshold. The analysis of ROC curve yielded an optimal threshold of 525 with the sensitivity rate of 96.1% (95% CI 0.868–0.989), specificity 89.7% (95% CI 0.815–0.945) and consistency 92.0% (95% CI 0.863–0.955), respectively. Youden index was 0.857 and AUC was 0.938 (95% CI 0.893–0.983) (Table 6). McNemar test showed no statistical difference between Crispr method and the gold sequencing. The Kappa value of Crispr method was 0.834, which manifested good consistency with direct sequencing method. The IHC was completely consistent with the direct sequencing results. The results of the IDH1-R132H tests in the typical cases were shown in Fig. S1.

**Table 6:** Consistency analysis of fresh sample groups between Crispr and direct sequencing method in IDH1-R132H detection.

Crispr	Gold standard		Total
	+	−	
+	49	9	58
−	2	78	80
Total	51	87	138

### Calibration of relative fluorescence value

**Figure 5:**
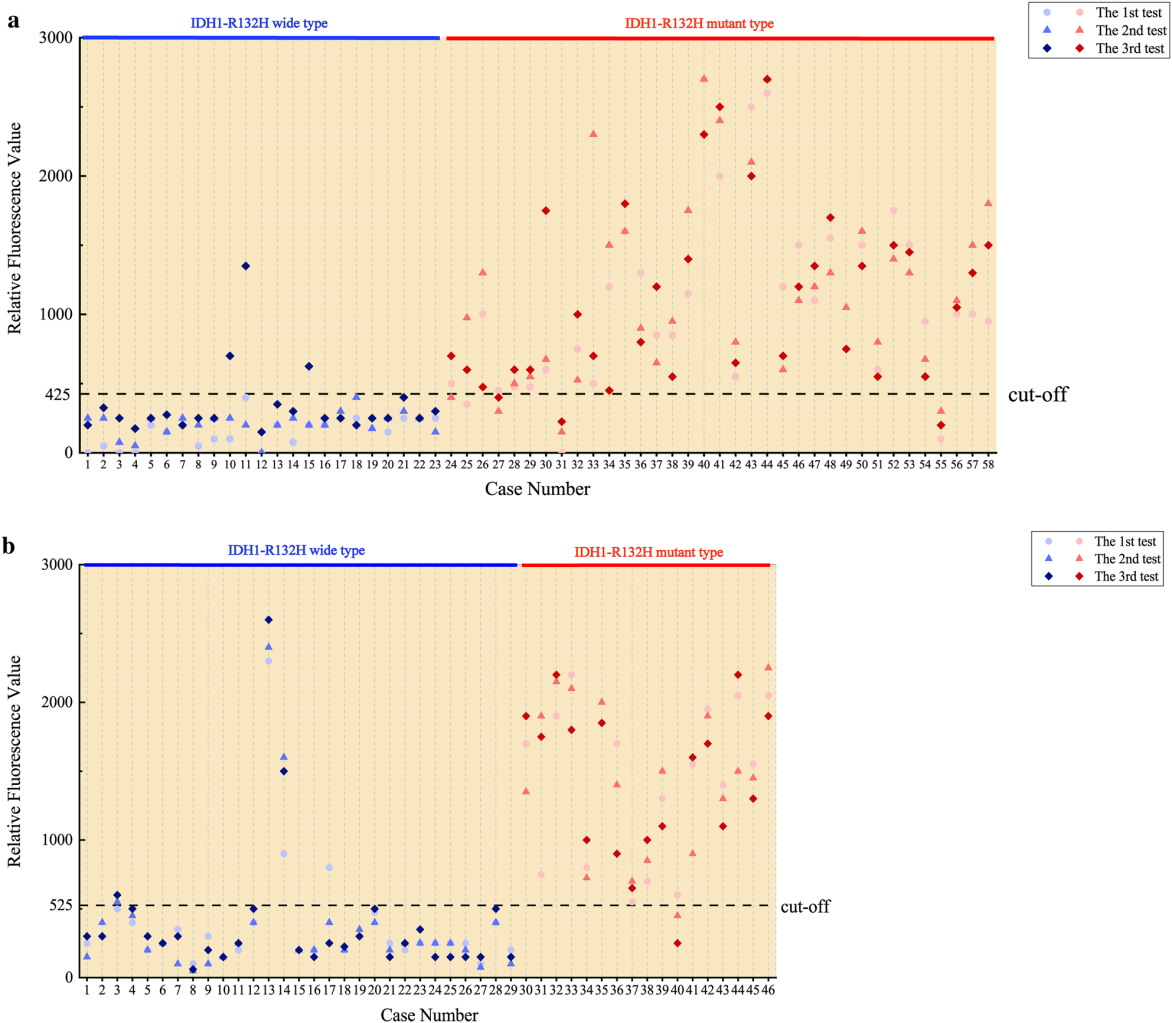
Scatter diagram of relative fluorescent value. (**a**) The relative fluorescent value of all samples in Group 1. No.10, No.11 and No.15 IDH1-wt samples had high relative fluorescent value, while No.8 and No.32 IDH1-R132H samples had low relative fluorescent value, which was not consistent with the gold standard. (**b**) The relative fluorescent value of all samples in Group 2. There was a significant difference between the relative fluorescent value of IDH1-wt and IDH1-R132H. No.13 and No.14 IDH1-wt samples and No.12 IDH1-R132H samples present abnormal relative fluorescent value. Fortunately, such errors were limited and tolerable (link: https://www.originlab.com/, version OriginPro 2018C).

We analyzed the differences of relative fluorescence value of IDH1-wt and IDH1-R132H between the two groups (Fig. 5). Due to the non-normal distribution of the data, we used the Man-Whitney U test. For IDH1-wt, the median relative fluorescence value of Group 1 and Group 2 was 250 (200, 400) and 250 (162, 250), respectively. The distribution of overall relative fluorescence value was statistically different between the two groups (z = − 2.348, *P* = 0.019). For IDH1-R132H, the median relative fluorescence value of Group 1 and Group 2 was 1000 (600, 1500) and 1500 (900, 1900), respectively. The distribution of overall relative fluorescence value was statistically different between the two groups (z = − 3.349, *P* = 0.001). The data were shown in Tables 7, 8.

**Table 7:** Comparison of relative fluorescence value in two groups.

	M (P_25_, P_75_)	Median difference (95% CI)	Mann–Whitney U Test	
			z value	P value
IDH1-wt				
Group 1	250 (200, 400)	50 (0–100)	− 2.348	0.019
Group 2	250 (162, 250)			
IDH1-R132H				
Group 1	1000 (600,1500)	400 (150–600)	− 3.349	0.001
Group 2	1500 (900,1900)

**Table 8:** Comparison of relative fluorescence value in two groups.

	M (P_25_, P_75_)	Median difference (95% CI)	Mann–Whitney U test	
			z value	P value
IDH1-wt				
Group 1	250 (200, 400)	50 (0–100)	− 2.348	0.019
Group 2	250 (162, 250)			
IDH1-R132H				
Group 1	1000 (600, 1500)	400 (150–600)	− 3.349	0.001
Group 2	1500 (900, 1900)			

## Discussion

In 104 glioma samples, we preliminarily finished the rapid detection of IDH1-R132H in 60 min using Crispr-Cas12a. The relative fluorescence value of IDH1-R132H was higher than IDH1-wt. In this assay, the intraoperative detection of Crispr-Cas12a shows comparable performance, which suggests that intraoperative diagnosis of IDH1-R132H can be realized with the current methodology.

IDH is one of the most important molecular markers of adult diffuse glioma, whose gene status is often closely associated with patient prognosis and can guide the resection range of glioma surgery and optimize the surgical strategy^1,10^. Today, the mainstream holds the opinion^1^ that for WHO grade 2–3 oligodendrogliomas with IDH mutations and 1p/19q co-deletion should focus on neurological protection during the operation because they are sensitive to chemoradiotherapy. Total resection at the expense of sacrificing functions was not recommended, and little tumor residues hardly have prognostic effect. While WHO grade 2–3 astrocytomas with IDH1-mut and IDH1-wt LGGs required the maximum safe resection beyond the abnormal range shown by MRI, even several milliliter tumor residues can bring a poor prognosis^4^. For WHO grade 4 gliomas, the astrocytomas with IDH1-mut benefited when resecting the non-contrast-enhancing (NCE) tumor on the basis of resecting the contrast-enhancing (CE) tumor. As for glioblastoma with IDH1-wt, the resection of NCE tumor seems not to improve the prognosis. The resection of CE tumor is recommended only for people over 65 years old, while patients under 65 years old can remove NCE tumor as much as possible on the condition of safety^5^.

Unfortunately, the way of obtaining IDH1 status is limited, and POC tests are not yet fully mature before or during the operation. Rapid IHC (R-IHC) can finish the test in half an hour, but it is a pity that no reagents for IDH detection have been reported^20^. Imaging-based machine learning model allows preoperative assessment of IDH1 status with poor stability for multicenter cases^21^. High-resolution melting is easy, fast and sensible, while it requires high quality for RT-PCR amplifier and operator^22^. There are also many detection methods for targeting 2-HG, such as the downstream metabolites of IDH-mut, including magnetic resonance spectroscopy (MRS)^23^, multiple mass spectrometry (MS)^24–26^ and microfluidics^27^. Among them, MS is relatively mature and can quickly and accurately detect IDH mutation in surgery, which is relying on expensive mass spectrometers. Thus, it is of great significance for us to explore a method of POC detection for IDH1. We preliminarily verified the feasibility of this method in IDH1-R132H detection using Crispr.

Currently, direct sequencing method is the well-recognized 'gold standard' for diagnosing IDH mutations, including Sanger sequencing method and NGS sequencing method. NGS sequencing method has been widely used in the diagnosis and treatment of clinical glioma patients with the advantages of high throughput, high accuracy and lower cost. The postoperative result of NGS sequencing is often combined with the molecular combination diagnosis. The IHC can detect the IDH1-mut protein with the fastest readout in about 3 h. It is a routine examination item for glioma and the preferred alternative to NGS sequencing and has high consistency with the result of NGS sequencing. Although these two detection methods have been quite well developed, the inability to perform intraoperative detection is their minor defect. Crispr is the 'star molecule' in recent years, which developed for gene editing in 2012^28^. Gene editing platforms based on Crispr/Cas have sprung up, and efficient and convenient nucleic acid testing tools such as DETECTR (based on Crispr-Cas12a) have derived^15^, which shows a significant advantage, such as high sensitivity and specificity, fast, low cost and multiple tests. The sensitivity is at amol (10^–18^) level, which enables to detect the coarse amplified samples and easily identifies the point mutation in the IDH1 (c.395G > A); mature Crispr/Cas tools and standard operation process can complete the qualitative detection in about 1 h^15,29^, and our experiment takes about 60 min, too; the cost of per reaction based on preamplifier system is less than 1 dollar without expensive reagents and instruments; CARMEN is a multiple method based on Crispr tool^30^, which can test more than 4500 kinds of nucleic acids at the same time, indicating the potential of Crispr-Cas12a detection. In terms of in vitro diagnosis, the applications of Crispr detection in Zika virus and dengue virus detection, HPV screening in cervical cancer and detection of EGFR mutation in non-small cell lung cancer are becoming increasingly mature. Therefore, the application of Crispr in the rapid molecular detection of diffuse glioma is extremely urgent. We innovatively used Crispr technology for rapid detection of IDH1-R132H with sensitivity and specificity exceeding 90%, which can be used to guide surgical strategies.

Among 80 percent of LGGs and secondary glioblastoma accompanied with IDH1-mut character are usually younger than people with IDH1-wt. The proportion of temporal lobe tumors is significantly higher in IDH1-wt glioma, which often has histopathological features of oligodendroglioma^8,31^. Combined with the postoperative molecular pathology, we found that about 1/5 of the patients required a revised diagnosis. For example, the cases which present oligodendroglioma in pathology without the molecular character such as IDH-mut and 1p/19q co-delection can’t be included in classical oligodendroglioma. WHO grade 4 tumors with IDH1-mut should mostly belong to astrocytoma, IDH1-mut and WHO grade 4 (exclude CDKN2A/B mutation). This phenomenon also reflects the necessity of genetic testing for adult diffuse glioma^1,7^. In addition, IDH mutation is one of the early events in adult diffuse glioma, which is the primary factor influencing molecular typing, regardless of grade^7^. However, it is difficult to judge molecular classification directly by imaging, and the accuracy of adult diffuse glioma molecular classification based on imaging characteristics is not satisfied^32^. Through the rapid intraoperative detection of IDH1-R132H locus mutation, combined with the tumor imaging characteristics, the operator can more accurately infer the molecular subtype and pathological classification of adult diffuse glioma, and then choose the best surgical strategy. WHO grade 2–3 adult diffuse gliomas often do not show significant enhancement, with only T2w and Flair high signal. For such tumors, if IDH1-R132H mutation is found by intraoperative Crispr-Cas12a test, and the tumor MRI shows sharp boundary, CT shows focal sheet calcification, and heterogeneous signal intensity in T2w images, it is more reasonable to diagnose oligodendroglioma^33^. At this time, the surgery should focus on the protection of nerve function. With sharp boundaries and T2/FLAIR-mismatch sign features, astrocytoma is more likely to diagnosis, and all tumors in T2w and Flair high signal areas need to be removed as much as possible^34^. If the IDH1-R132H mutation is not detected, and the tumor boundary is blurred and the invasive and growth characteristics are prominent, IDH wild-type adult diffuse glioma should be diagnosed, and more radical surgical resection may be required^35^. It is worth mentioning that, for some non-neoplastic lesions, such as inflammatory pseudotumors, they often do not have the molecular characteristics of IDH mutation^36^. Intraoperative monitoring of IDH1-R132H mutations with Crispr-cas12a can help distinguish these lesions from adult diffuse gliomas.

In this study, we found that only 1 had the contradictory outcome between IHC and direct sequencing method, and the results of ddPCR (without IDH1-R132H) of 2 cases differed from direct sequencing method (with IDH1-R132H). It was explicable that intraoperative sampling was inaccurate, or the heterogeneity of glioma, although it has been reported that IDH1 mutations are early, stable molecular events in glioma^8,37–39^. We hold the opinion that the two cases should be considered as IDH1-wt despite the results of direct sequencing method. We regarded the result of direct sequencing method as gold standard and took the results of ddPCR into account at the same time. For the operation of Crispr detection, all the tests of samples were performed by the same two inspectors using the same rapid fluorescence detector. Besides, we innovatively used the direct amplification tube which needed not to be opened again during the test after two sampling additions. The detection reagents of the upper tube can be directly dumped into the lower tube and mixed with the amplification reagents to avoid aerosol contamination. In conclusion, our experiment has a good quality control. In addition, we found that compared with previous frozen samples, the relative fluorescence value of IDH1-R132H samples was significantly higher, and the interference by IDH1-wt also seemed to be more obvious in recent fresh samples. Although it was previously reported that long-term cryopreservation of tumor tissues was placed at – 80 °C or even that repeated freeze–thaw cycles did not have a significant effect on DNA concentration^40,41^. Therefore, we analyzed the relative fluorescence value thresholds of the two groups separately, believing that the threshold of IDH1-R132H and IDH1-wt in Group 2 should be a little higher. This suggested that the threshold should be higher when Crispr-Cas12a was applied to intraoperative detection.

There are also many issues required for improvement in our experiment. First, we did not complete the quantitative test, but the lack of quantitative data did not affect the intraoperative results. Secondly, we only focused on the mutations at R132H and ignored other locus such as IDH2, which although has a low probability of occurrence. The detection of other locus can be developed in the future. Finally, the reactive time we cost (about 60 min) is slightly long for craniotomy, so the detection process and reagents still need to be further optimized.

## Conclusion

This experiment used Crispr-Cas12a to achieve the rapid detection of IDH1-R132H. The preliminary results showed that the method has high sensitivity, specificity and consistency rate and basically achieved the rapid qualitative detection, which has the potential value in clinical application.

## Supplementary Information

Supplementary Legends.

Supplementary Figure S1.

## Data Availability

The datasets used and/or analyzed during the current study are available from all the authors. The datasets generated and/or analysed during the current study are available in the OMIX repository, https://ngdc.cncb.ac.cn/omix/release/OMIX002838.
