# Supplementary material for: Rapid detection of isocitrate dehydrogenase 1 mutation status in glioma based on Crispr-Cas12a
Source: Sci Rep. 2023 Apr 7;13:5748. doi: 10.1038/s41598-023-32957-y (PMC10081818; doi:10.1038/s41598-023-32957-y)
Supplement: Supplementary file 1 — Supplementary Legends. [file 41598_2023_32957_MOESM1_ESM.docx]

**Figure S1. Classic cases of IDH1-R132H and IDH1-wt.**

**(a and b)** Samples collection with the assistance of intraoperative MRI navigation system. **a.** The postoperative pathology was astrocytoma, WHO grade 4, IDH1-R132H. **b.** The postoperative pathology was glioblastoma, WHO grade 4, IDH-wt. **(c, e, g)** The results of detecting the IDH mutations in this sample by IHC, Crispr-Cas12a and NGS sequencing method, respectively. **(d, f, h)** The results of detecting the IDH mutations in this sample by IHC, Crispr-Cas12a and NGS sequencing method, respectively.
