# Supplementary figures and images for: Rapid detection of isocitrate dehydrogenase 1 mutation status in glioma based on Crispr-Cas12a
Source: Sci Rep. 2023 Apr 7;13:5748. doi: 10.1038/s41598-023-32957-y (PMC10081818; doi:10.1038/s41598-023-32957-y)

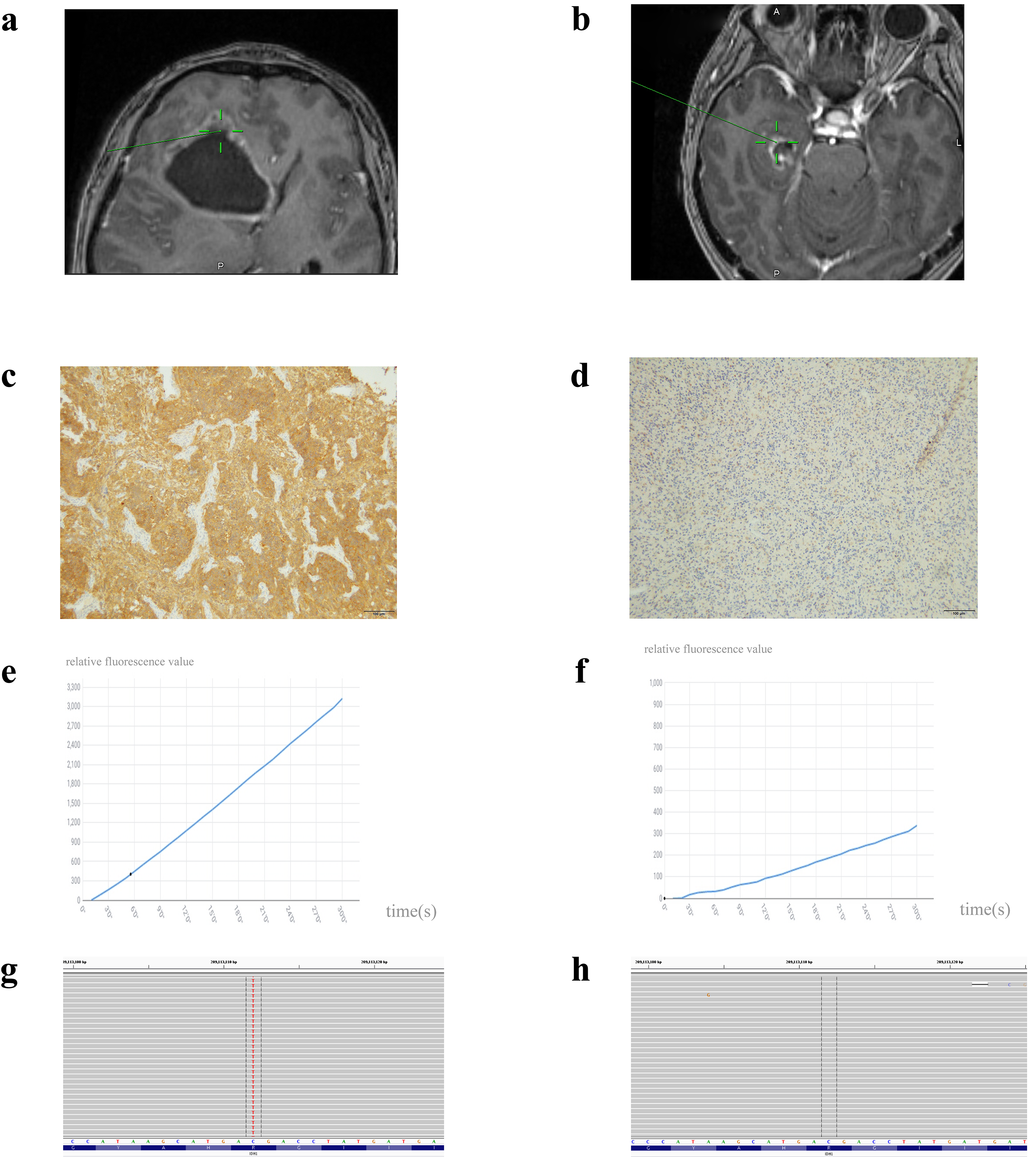

Supplement: Supplementary file 2 — Supplementary Figure S1. [file 41598_2023_32957_MOESM2_ESM.tif]
